# A Screen for F1 Hybrid Male Rescue Reveals No Major-Effect Hybrid Lethality Loci in the *Drosophila melanogaster* Autosomal Genome

**DOI:** 10.1534/g3.114.014076

**Published:** 2014-10-27

**Authors:** Tawny N. Cuykendall, P. Satyaki, Shuqing Ji, Derek M. Clay, Nathaniel B. Edelman, Alexandra Kimchy, Ling-Hei Li, Erin A. Nuzzo, Neil Parekh, Suna Park, Daniel A. Barbash

**Affiliations:** Department of Molecular Biology and Genetics, Cornell University, Ithaca, New York 14853

**Keywords:** hybrid incompatibility, shmIRs, speciation

## Abstract

Hybrid sons between *Drosophila melanogaster* females and *D. simulans* males die as 3rd instar larvae. Two genes, *D. melanogaster Hybrid male rescue (Hmr)* on the X chromosome, and *D. simulans Lethal hybrid rescue (Lhr)* on chromosome II, interact to cause this lethality. Loss-of-function mutations in either gene suppress lethality, but several pieces of evidence suggest that additional factors are required for hybrid lethality. Here we screen the *D. melanogaster* autosomal genome by using the Bloomington Stock Center Deficiency kit to search for additional regions that can rescue hybrid male lethality. Our screen is designed to identify putative hybrid incompatibility (HI) genes similar to *Hmr* and *Lhr* which, when removed, are dominant suppressors of lethality. After screening 89% of the autosomal genome, we found no regions that rescue males to the adult stage. We did, however, identify several regions that rescue up to 13% of males to the pharate adult stage. This weak rescue suggests the presence of multiple minor-effect HI loci, but we were unable to map these loci to high resolution, presumably because weak rescue can be masked by genetic background effects. We attempted to test one candidate, the dosage compensation gene *male specific lethal-3 (msl-3)*, by using RNA interference with short hairpin microRNA constructs targeted specifically against *D. simulans msl-3* but failed to achieve knockdown, in part due to off-target effects. We conclude that the *D. melanogaster* autosomal genome likely does not contain additional major-effect HI loci. We also show that *Hmr* is insufficient to fully account for the lethality associated with the *D. melanogaster* X chromosome, suggesting that additional X-linked genes contribute to hybrid lethality.

Speciation requires the evolution of reproductive isolating barriers that prevent the production of viable and fertile offspring between groups of individuals. Several isolating mechanisms maintain these barriers and are classified as either premating or postmating. Hybrid incompatibility (HI), the lethality and sterility of interspecific hybrid progeny, is an example of the latter. The Dobzhansky-Muller (D-M) model posits that HI is an indirect consequence of lineage-specific evolution and arises from negative epistatic interactions among alleles in the hybrid background (Maheshwari and Barbash 2011). The simplest form of the D-M model invokes two loci. For example, the ancestral genotype *a_1_a_1_b_1_b_1_*, where *a_1_* and *b_1_* are the ancestral alleles of genes *a* and *b*, diverges between two independently evolving lineages, whereby each lineage fixes a new allele, giving rise to two derived genotypes, *a_2_a_2_b_1_b_1_* and *a_1_a_1_b_2_b_2_*. The incompatible interaction arises between the ‘*a_2_*’- and the ‘*b_2_*’-derived alleles in the hybrid. A fundamental question arising from this model is whether HI is caused by a simple interaction between 2 loci, as illustrated by this general example of a D-M interaction, or rather by complex multilocus interactions.

Matings between *Drosophila melanogaster* females and *D. simulans* males produce sterile hybrid females and invariantly lethal hybrid sons, which die as 3rd instar larvae (Barbash 2010b). Brideau *et al.* (2006) showed that hybrid lethality in this cross is due in part to the epistatic interaction between the genes *Hybrid male rescue* (*Hmr*), on the *D. melanogaster* X chromosome, and *Lethal hybrid rescue* (*Lhr*), on the *D. simulans* second chromosome, in a manner consistent with the D-M model (Brideau *et al.* 2006). *Hmr* and *Lhr* are characterized as major-effect HI genes because loss-of-function mutations in *D. melanogaster Hmr* or *D. simulans Lhr* suppress hybrid male lethality (Hutter and Ashburner 1987; Watanabe 1979). Both *Hmr* and *Lhr* are evolving rapidly due to positive selection in both the *D. melanogaster* and *D. simulans* lineages, suggesting functional divergence of the orthologs (Brideau *et al.* 2006; Maheshwari *et al.* 2008). Rescue by *Lhr* is asymmetric; only elimination of *D. simulans Lhr* rescues lethality to produce viable adult males, suggesting functional divergence of the *Lhr* coding sequence with respect to hybrid lethal activity (Brideau *et al.* 2006).

However, functional divergence of *Lhr* is more complex than originally proposed based on its asymmetry of rescue. Transgenic lines of *D. melanogaster* expressing either *D. melanogaster* or *D. simulans Lhr* transgenes were generated, and the hybrid lethal activity of each ortholog was assayed by testing for complementation (*i.e.*, suppression) of the *D. simulans Lhr^1^* hybrid rescue mutation (Maheshwari and Barbash 2012). Despite their extensive sequence divergence, both transgenes suppressed rescue, indicating that hybrid lethal activity is an ancestral function of *Lhr* (Maheshwari and Barbash 2012). Further experiments showed that *D. melanogaster Lhr* is expressed at a lower level in hybrids compared with *D. simulans Lhr*, suggesting that *D. simulans Lhr* may have greater hybrid lethal activity because it is expressed at a greater level in hybrids. Consistent with this interpretation, two *D. melanogaster Lhr*^–^ deletions produced weak rescue to the pharate male stage (7–21% of total deficiency-carrying progeny) (Maheshwari and Barbash 2012).

Although *Hmr* and *Lhr* are major-effect hybrid lethality genes, additional factors likely contribute to lethality. Experiments performed by Muller and Pontecorvo nearly 70 years ago (1940, 1943) suggest that F1 hybrid male lethality involves interactions between loci on the *D. melanogaster* X (*Hmr*), the *D. simulans* 2nd chromosome (*Lhr*), and the *D. simulans* 3rd chromosome (Muller and Pontecorvo 1940; Pontecorvo 1943). More recently, Brideau *et al.* (2006) found that expression of *D. simulans Lhr* in a *D. melanogaster* background is not lethal, demonstrating that the interaction between *D. melanogaster Hmr* and *D. simulans Lhr* is insufficient to cause lethality (Brideau *et al.* 2006). Taken together, these studies strongly suggest that additional factors contribute to lethality.

Several screens of the *D. melanogaster* genome have searched for additional HI genes in *D. melanogaster/D. simulans* hybrids (Coyne *et al.* 1998; Presgraves 2003; Matute *et al.* 2010). Coyne *et al.* (1998) crossed *D. melanogaster* stocks containing deficiencies (deletions) to *D. simulans* males and assayed F1 hybrid female viability. This screen was designed to identify genes that cause lethality when hemizygous in a hybrid background. These regions could potentially contain genes that are haploinsufficient or recessive lethal in hybrids. The screen covered just less than 50% of the *D. simulans* genome and did not find any regions that caused unconditional lethality. Matute *et al.* (2010) repeated this screen with coverage increased to 79.4% of the genome and identified 10 regions that cause lethality when hemizygous in hybrid females (Matute *et al.* 2010). Presgraves (2003) performed a more sensitive screen by crossing *D. melanogaster* females with *D. simulans* males carrying the *Lhr* hybrid rescue mutation and assaying the viability of rescued F1 hybrid males (Presgraves 2003). This screen, unlike that of Coyne *et al.* (1998), can identify recessive-recessive interactions between the X chromosome and the autosomes. He screened ~70% of the *D. simulans* genome and found 40 nonoverlapping regions (20 lethal, 20 semilethal), that when hemizygous in hybrids, cause lethality in rescued males, concluding that recessive-recessive HI is the most common type of interaction. The genes mapped within these regions include two nucleoporins, *Nup96* (Presgraves 2003) and *Nup160* (Tang and Presgraves 2009).

However, the genes identified in the aforementioned screens act recessively and are therefore not expected to affect F1 hybrid male viability. In contrast, both *Hmr* and *Lhr* are dominant and their presence causes lethality in the F1 generation. Here we use the Bloomington Deficiency Kit to systematically screen the vast majority of the *D. melanogaster* autosomal genome for genes with effects similar to *Hmr* and *Lhr* by crossing deficiency-carrying females to *D. mauritiana* males (Figure 1). *Hmr* mutations rescue hybrid males with all three sibling species of *D. melanogaster* but rescue best with *D. mauritiana* compared with *D. simulans* and *D. sechellia* (Hutter and Ashburner 1987). Because *D. mauritiana* and *D. simulans* are very closely related, having diverged within the past 250,000 years (Kliman *et al.* 2000; McDermott and Kliman 2008), we expect that most hybrid lethality genes are shared between the two species, but it is possible that lineage-specific HIs have evolved. For example, *Nup96*-dependent lethality is specific to *D. simulans* and *D. sechellia* (Barbash 2007). To further increase sensitivity, we screened for rescue to the pharate adult stage, because *D. melanogaster* deletions of *Lhr* rescue to this stage (Maheshwari and Barbash 2012). The difference in strength of rescue when deleting *D. melanogaster vs. D. simulans Lhr* appears to be due to a greater expression level of *D. simulans Lhr* in hybrids (Maheshwari and Barbash 2012). Based on these previous findings with *Lhr*, we suggest that our screen also has the potential to uncover genes where the *D. mauritiana* (or *D. simulans*) allele contributes more to hybrid lethality than the *D. melanogaster* allele.

**Figure 1:**
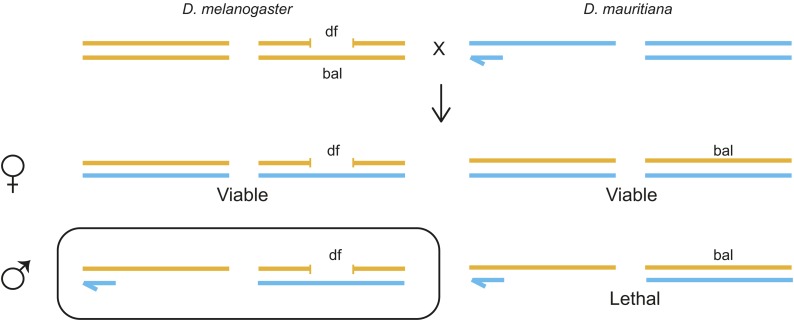
Screen design. *D. melanogaster* females from the Bloomington Deficiency Kit were crossed to *D. mauritiana* males. Female progeny inheriting either the deficiency chromosome (df) or the balancer chromosome (bal) are viable, though sterile. Males inheriting the balancer chromosome are invariably lethal. However, if the deficiency deletes a hybrid lethality gene we expect to observe rescue of this class of males (circled). We consider rescue to be survival to the pharate adult stage or beyond.

## Materials and Methods

### Fly stocks and crosses

*D. melanogaster* female flies from the Bloomington Deficiency Kit were crossed to at least two different lines of *D. mauritiana* (Figure 1). The *D. mauritiana* lines included *w^1^f^2^*, W139, and two different isofemale lines (105 and 207). Crosses were set up at 18° with ~20 females and ~25 males, flipped every 3−4 d for 2 wk, and progeny scored until the last fly eclosed. Pharates were then dissected to determine sex and, where possible, genotype. Rescue was calculated by dividing the number of rescued male pharates by the number of females carrying the deficiency chromosome. A *D. melanogaster Lhr* deletion stock (*Df(2R)k08901*, which we will refer to as *Df(2R)Lhr*^–^), was used as a positive control for pharate rescue (Maheshwari and Barbash 2012).

### Genome coverage

The proportion of the genome covered by the screen was calculated based on either the known or estimated molecular breakpoints of the deficiencies. Approximately 40% of the deficiencies are not mapped molecularly. For these, we estimated cytological breakpoints using GBrowse on FlyBase and *D. melanogaster Gene Models/Evidence* (*R5.48*) to convert to molecular estimates. When there was uncertainty in the cytological location of a breakpoint, we took the average of the extremes of the described range. We then determined regions of overlap among deficiencies and counted each region (*i.e.*, base pair) only once to arrive at the total number of base-pairs screened. This was done for each chromosome arm separately and then divided by the total number of base-pairs in the chromosome to determine the percentage of the chromosome arm covered.

### RNAi construct design and knockdown

We used the short hairpin microRNA (shmiR) system of RNA interference (RNAi) with the Valium20 vector to attempt to knock down gene expression in hybrids (Haley *et al.* 2008; Ni *et al.* 2011). We initially designed 21-bp siRNAs, either with a single mismatch at position 2 (thought to ensure that only the antisense siRNA is loaded into the slicer complex), or without any mismatches as in (Ni *et al.* 2011) (Table 1). After three constructs failed to knockdown *D. simulans msl-3 (sim-msl-3)*, we made several design modifications. First, we increased the small interfering RNA (siRNA) length to 22 bp because most short hairpin RNAs (shRNAs) made from three different shRNA constructs expressed in S2 cells were 22 bp in length (Ni *et al.* 2011), and most microRNAs from the endogenous miR1 locus are also 22 bp (Ruby *et al.* 2007). Additionally, 22 bp siRNAs were found to have better silencing than shorter siRNAs (Wu *et al.* 2011). Second, mismatches between the guide and passenger strands were included at positions 2 and 11 (Haley *et al.* 2008) to mimic the endogenous structure of miR1. Note, however, that sequencing data suggest that mismatches are not necessary to achieve preferential accumulation of the guide *vs.* passenger strands (Ni *et al.* 2011). A, C and G were mismatched with the same nucleotide; U was mismatched with C (Wu *et al.* 2011).

**Table 1:** ShmIR constructs and summary of results

Construct	Length	Mismatches	AS Sequence	Knockdown Assayed by RT-PCR?; Result
*sim-Lhr-shRNA-577*	21	Mismatch at bp 2	TAGATTCATTGCTAACACCAT	Yes; knockdown
*sim-msl-3-shRNA-579*	21	Mismatch at bp 2	TATTGTGATAGAAGGTCTCGG	Yes; no knockdown
*sim-msl-3-shRNA-599*	21	None	TAACATAGTTCTCCCTGTCGA	N/A; lethal to both sexes in *D. melanogaster*
*sim-msl-3-shRNA-600*	21	None	TAGTACCTTGACCATATTCCG	Yes; lethal to *D. melanogaster* males
*sim-msl-3-shRNA-601*	21	None	TAGCGCCGTCATCACTTGCAG	Yes; no knockdown
*sim-msl-3-shRNA-633*	22	Mismatches at bp 2 and 11	TGAATGGGACCAAGTTAGTCAC	N/A; lethal to both sexes in *D. melanogaster*
*sim-msl-3-shRNA-634*	22	Mismatches at bp 2 and 11	TCTCCCGTGTGGAGTGGATCCA	Yes; no knockdown.
*sim-msl-3-shRNA-635*	22	Mismatches at bp 2 and 11	TCGCACATGGGCATCGACCGAT	N/A; semilethal to both sexes in *D. melanogaster*

ShmIR, short hairpin microRNA; AS, anti-sense; RT-PCR, reverse-transcription polymerase chain reaction; N/A, not applicable.

### Reverse-transcription polymerase chain reaction (RT-PCR):

cDNA synthesis was performed as in Maheshwari and Barbash (2012). RT-PCR for *Lhr* used the primers GTAGCTTTCTCTTGGCGCTCTT and GTAAGTGAACTGAAGCTGCGTTGG, which span a fixed indel between *D. melanogaster* and *D. simulans Lhr*, amplifying products of 278 bp and 326 bp from *D. melanogaster* and *D. simulans*, respectively. RT-PCR for *D. melanogaster msl-3 (mel-msl-3)* used the primers AGGAAAAACCCCCGTCCGGA and GCGTGCTGTTTGCCTAGTACCTT. RT-PCR for *sim-msl-3* used the primers AGGAGAAACCCCCGCCACCC and GCGTGCTGTTTGCCTAGTACCTT.

### Molecular breakpoint determination

*Df(3L)BSC27* (6867) was outcrossed to a sequenced wild-type DGRP strain (NC486). *Df(3L)BSC33* (6964) is balanced over *TM2*, and we did not outcross this stock to NC486 because *Ubx* was not sufficiently expressed to accurately genotype the progeny. DNA was isolated from 3-day-old females (n = 10) using the QIAGEN DNeasy Blood and Tissue Kit according to the supplemental insect protocol. The Epigenomics Core Facility at Weill Cornell Medical Center constructed the libraries and performed paired-end sequencing.

Raw reads (50 bp) were aligned separately to the reference genome (Dmel r5) using the default settings of BWA 0.6.2. Three different methods were used to analyze the sequences: split-read mapping, paired-end mapping, and depth of coverage. The split-read mapping was performed by Pindel (Ye *et al.* 2009), which identifies paired reads in which only one read maps to the reference genome. This mapped read serves as an anchor point for the detection of the other read. Depending upon the value of the expected sizes of copy number variants (CNVs), Pindel then splits the unmapped read into two or three fragments and maps them separately. Paired-end analysis was performed using Hydra and Delly (Quinlan *et al.* 2010; Rausch *et al.* 2012), which identify read-pairs that do not map to the reference genome with the expected size and orientation and then clusters them based on which CNV they support. This approach successfully identified the deletion in *Df(3L)BSC27*. Finally, depth of coverage was assessed using BEDTools to calculate the coverage per 50-bp interval and plotted in R. Breakpoints can be determined to within 500 bp with this method, which confirmed the predicted breakpoints in *Df(3L)BSC33*. Illumina sequence data are available from the NCBI website under the study accession SRP044233.

## Results

### *D. melanogaster Lhr* rescues F1 hybrid male lethality

We recently reported that *D. melanogaster Lhr (mel-Lhr)* has weak hybrid lethal activity (Maheshwari and Barbash 2012). Two different *Lhr*^–^ deletions produced ~7–21% rescue of males to the pharate adult stage in crosses to *D. mauritiana* (Maheshwari and Barbash 2012). We therefore used *Df(2R)Lhr*^–^ as a positive control for hybrid male rescue in our screen and observed rescue with two different stocks of *D. mauritiana*, averaging 7–8% at 18° (Table 2).

**Table 2:** Genome coverage of screen

Arm	All Stocks Screened	Sensitivity	
		≥50 Df-Carrying Females Obtained	≥100 Df-Carrying Females Obtained
2L	92.4%	52.8%	21.9%
2R	81.1%	56.3%	23.9%
3L	86.5%	83.8%	61.9%
3R	94.0%	91.2%	91.0%
Total of chromosomes 2 and 3	88.9%	72.5%	52.4%

However, as reported in Maheshwari and Barbash (2012), we also found that other *Lhr*^–^ deletions (*Df(2R)BSC44* and *Df(2R)BSC161*), did not rescue males to the pharate stage. We conclude that our screen is vulnerable to false negatives due to background effects in the genome that affect our ability to detect weak rescue. This finding complicates mapping efforts because stocks that do not rescue are uninformative.

### Lack of evidence for additional major-effect HI loci in the *D. melanogaster* genome

We screened 278 stocks from the Bloomington Deficiency Kit, covering approximately 89% of the autosomal genome (Supporting Information, Table S1). The average number of female progeny per cross was 238, with a slight bias for progeny carrying the deficiency chromosome (*t*-test, *P* < 0.001). However, the numbers varied widely among crosses due to variable mating efficiency. A total of 72.5% and 52.4% of crosses produced a minimum of 50 and 100 deficiency-carrying females, respectively, summed over replicates. The percentage of each chromosome arm covered is shown in Table 2.

We did not find any regions in the *D. melanogaster* genome, which, when removed, produce viable hybrid males (a single live male was found in one replicate with *Df(3L)XDI98* but not in three other replicates). Therefore, within the regions of the genome that we screened, there are likely no additional major-effect *D. melanogaster* hybrid lethality genes. However, we found two adjacent deficiencies on 3L that give rescue comparable to positive controls with *Lhr*^–^ deficiencies, *Df(3L)BSC27* (65D4-65E6) and *Df(3L)BSC33* (65E10-65F6) (Table 3). We also found weak rescue with deficiencies spanning 75A6-76D5 (Table 3). Further characterization of these two regions is described in the following sections. Three additional regions, two on 3L and one on 3R, also produced pharate males but were not pursued for further study due to low or variable rescue and/or the paucity of additional deficiency stocks (Table 3). For example, *Df(3L)emc-E12* produced 6.7% rescue with *D. mauritiana* W139 but no rescue with strain iso 105. Thus, we conclude that rescue to the pharate stage is a rare event and is vulnerable to background effects.

**Table 3:** Regions that rescue hybrid males to the pharate adult stage

Region	Deficiency (% Rescue)	Molecular Breakpoints*^a^*		Inferred Molecular Breakpoints*^b^*	
*Lhr*	Df(2R)BSC49 (21.4%)			12738807	13290649
	Df(2R)k08901 (7.21%/8.31%)	13309963	13340212		
	Df(2R)BSC44			13166788	13309036
	Df(2R)BSC161	13192288	13372333		
61A-62E5	Df(3L)emc-E12 (6.7%)			206780	885293
	Df(3L)R-G7 (3.0%)			1863545	2541764
	Df(3L)Ar14-8			641337	1615040
	Df(3L)BSC181	1688724	1841694		
65D-66C5	Df(3L)BSC27 (5.6%/10.8%)	6935985	7149104		
	Df(3L)RM5-2*^c^*			6999777	7879617
	Df(3L)BSC33 (12.8%)	7271620	7319021		
	Df(3L)GN24			3922651	5203390
	Df(3L)XDI98			3967594	4134155
	Df(3L)ZN47			5096316	6696471
	Df(3L)W5.4			5919622	7029849
	Df(3L)BSC411	5969060	6618726		
	Df(3L)Exel6109	6736213	6936639		
	Df(3L)BSC224	6957557	7150109		
	Df(3L)BSC374	6957558	7032145		
	Df(3L)RM5-1			6999777	7287396
	Df(3L)Exel6110	7087906	7149284		
	Df(3L)BSC117	7242575	7328086		
	Df(3L)pbl-X1			7349893	8129687
	Df(3L)Exel8104	7353086	7522363		
	Df(3L)ZP1			7889239	8254722
75A6-76D5	Df(3L)W10 (1.9%)			17867203	18202039
	Df(3L)fz2 (1.7%)			19148197	19226562
	Df(3L)BSC20 (0.8%)			19360266	19492579
	Df(3L)kto2 (3.5%)			19380732	19924632
	Df(3L)BSC8			17656096	18009745
	Df(3L)Cat			18056276	18834273
	Df(3L)ED4782	18988994	19163802		
	Df(3L)XS533			19481010	20314886
83B7-83D1	Df(3R)BSC47 (3.9%/1.7%)			1509535	1756808
	Df(3R)BSC464	1474083	2037668		

In cases in which rescue occurred with both strains, percent rescue with *D. mauritiana* W139 is presented first, followed by percent rescue with *D. mauritiana* iso 105. Overlapping deficiencies that do not rescue also are listed.

a Breakpoints molecularly mapped.

b Breakpoints inferred based on cytology.

c *Df(3L)RM5-2* produced pharate hybrid males in initial crosses, but further testing failed to reproduce this result. See Table S1.

### Characterization of 75A-76D region

The initial cross with *Df(3L)W10* produced 7.5% hybrid pharate males with *D. mauritiana* iso 105, but additional replicates failed to produce rescue. Summing across replicates yields a final rescue of 1.9%. Two deficiencies, *Df(3L)BSC8* and *Df(3L)Cat*, which when combined encompass all of *Df(3L)W10*, did not rescue. However, other deficiencies adjacent to *Df(3L)W10* did rescue. *Df(3L)fz2*, which is ~80 kb and predicted to be ~1 Mb distal to *Df(3L)W10* rescued males at a similar low level (1.7%). Two additional deficiencies, *Df(3L)BSC20* and *Df(3L)kto2*, ~130 kb distal to *Df(3L)fz2* gave weak rescue (0.8% and 3.5%, respectively). These results suggest the possibility of multiple minor-effect genes in the 75A-76D region, but the low and variable level of rescue precluded further mapping.

### Characterization of the 65D-E region

**Figure 2:**
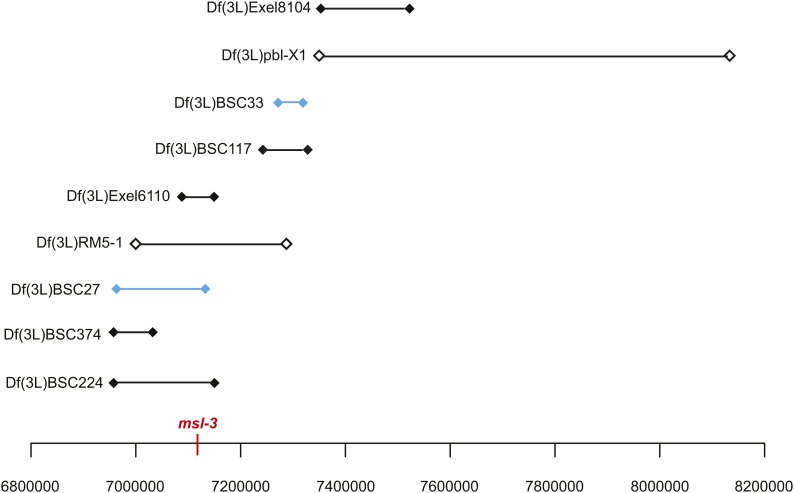
Deficiencies screened spanning 64C-66A. *Df(3L)BSC27* and *Df(3L)BSC33* spanning 65D4-65F6 produced rescued hybrid male pharates when crossed to *D. mauritiana* (blue bars). Seven deficiencies spanning this region that did not rescue are also shown. Filled endpoints denote molecularly defined deletions, whereas open endpoints indicate estimated breakpoints. Deficiencies *Df(3L)Exel6110* and *Df(3L)BSC27* were tested for complementation with *msl-3^1^*; neither complemented *msl-3^1^*, consistent with their molecularly mapped breakpoints. Complementation results are presented in Table S2.

The *Df(3L)BSC27* and *Df(3L)BSC33* deletions both rescued pharate males greater than 10%. They are predicted to be 213 kb and 96 kb, respectively, and to be separated by 122 kb (Figure 2). When crossed together, we found *trans*-heterozygotes are viable, which is consistent with nonoverlapping deficiencies. These results suggest either that there are two hybrid lethality loci in the region, or that they affect a single gene that is between them.

None of 13 additional deficiencies in this region produced pharate males (Figure 2 and Table 3). The nonrescuing deficiencies include *Df(3L)BSC224*, which deletes all but 22 kb of *Df(3L)BSC27*, and *Df(3L)BSC117*, which encompasses the rescuing *Df(3L)BSC33*. One possibility is that the rescuing deletion stocks contain second-site mutations that are responsible for the rescue. However, considering our aforementioned results showing variable rescue with *Lhr*^–^ deletions, we conclude that *Df(3L)BSC27* and *Df(3L)BSC33* are identifying two candidate regions but that our power to further map the hybrid rescue gene(s) is severely limited by false negatives.

### Characterization of molecular breakpoints by Next-Generation Sequencing of rescuing 3L deficiencies

**Figure 3:**
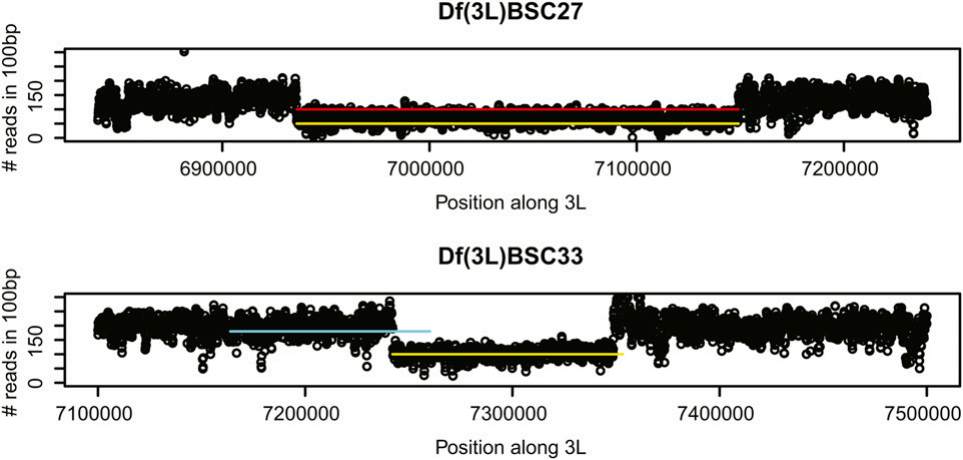
Coverage plots of deficiency stocks based on sequencing data. Coverage analysis supports the predicted deletions for *Df(3L)BSC27* and *Df(3L)BSC33*. The number of reads mapping within 100-bp intervals is plotted against the corresponding position on 3L for the sequenced deficiency lines. Segments in yellow represent the predicted location of deletions based on previous molecular or cytological estimates. Segments in red and cyan represent deletions predicted by paired-end approaches (Hydra and/or Delly) and Pindel, respectively.

We attempted to confirm the breakpoints of the rescuing deletions by short-read sequencing. We identified the breakpoints of *Df(3L)BSC27* using both Hydra and Delly, which use paired-end read analysis (Quinlan *et al.* 2010; Rausch *et al.* 2012). The breakpoints were identified to be within 200 bp of those reported previously (Table 3). Pindel, which uses split-read analysis, did not identify this deletion, likely due to the 50-bp read length; however, our analysis of read-depth further supported this deletion (Figure 3). Neither paired-end nor split-read analysis determined the correct deletion in *Df(3L)BSC33*. Pindel identified a deletion partially overlapping the predicted region and matching the predicted size but it was not supported by read-depth analysis (Figure 3). However, read-depth analysis did identify a deletion that corresponds well to the predicted molecular breakpoints previously reported (Table 3). We conclude that the *Df(3L)BSC27* and *Df(3L)BSC33* deletion stocks are correct.

Sixty-three genes are annotated within *Df(3L)BSC27* and *Df(3L)BSC33* (Table 4). We prioritized candidates based on shared characteristics with *Hmr* and *Lhr*, such as encoding proteins that are nuclear, rapidly evolving, highly expressed in ovaries, chromatin-binding, contain MADF and/or BESS domains, and are heterochromatic. Seven genes (*tow*, *msl-3*, *Mis12*, *cdc27*, *bin*, *MED4*, and *mei-P22*) encode nuclear proteins involved in processes including dosage compensation, mitosis, transcription, and meiotic recombination. The msl-3 protein contains a chromatin-binding chromo domain (Koonin *et al.* 1995) as well as an MRG domain that is implicated in chromatin remodeling (Bertram and Pereira-Smith 2001), whereas bin has a forkhead DNA-binding domain (Pérez Sánchez *et al.* 2002). Mis12 localizes specifically to the kinetochore and functions in mitotic spindle formation (Goshima *et al.* 2007). CG9948 is largely uncharacterized but is of interest because it contains a MADF domain, similar to Hmr. Five of the candidate genes encode proteins highly expressed in the ovaries (*RpL18*, *CG9953*, *Cdc27*, *Galphai*, and *sgl*) with functions that include translation, proteolysis, mitosis, receptor binding, and oxidoreductase activity.

**Table 4:** Genes mapped within rescuing deficiencies

Deficiency	No. Genes (High-Priority Candidates)	Genes*^a^*
*Df(3L)BSC27*	44 (9)	***tow***, ***msl-3***, ***Cpr65Ec***, *CG17744*, *CG10077*, *Surf1*, *corn*, *form3*, *Cpr65Eb*, *mp*, ***Mis12***, *melt*, ***Galphai***, ***CG9953***, ***CG9948***, *CR32385*, *CG10063*, *CG34030*, *CG43439*, ***bin***, *CG14823*, *CG10075*, *CG32391*, *CG8629*, *Cpr65Ea*, *CG15829*, *CG8641*, *CG8628*, *CG32388*, *BBS1*, *Dbi*, *CG10064*, ***sgl***, *Prat2*, *ms(3)04202*, *mp*, *Me*, *Vn*, *CS3-1*, *rip*, *dv*, *E(Ubx)3L*, *jv*
*Df(3L)BSC33*	21 (4)	***MED4***, *unc-13-4A*, *mRpL50*, *CG14830*, *Neos*, *CG14829*, *CR43470*, ***mei-P22***, *Dscam2*, ***RpL18***, *CG14826*, *BHD*, ***Cdc27***, *ms(3)04202*, *CS3-1*, *anon-65Ea*, *CG8628*, *Dscam2*, *corn*, *E(Ubx)3L*, *form3*

a High-priority candidates are indicated in bold, using criteria described in the section *Results*.

We focused on the gene *male specific lethal-3* (*msl-3*) in part because, like *Hmr* and *Lhr*, it encodes an adaptively evolving chromatin-binding protein (Rodriguez *et al.* 2007). The msl-3 protein is part of the dosage compensation complex (DCC) that binds to the X chromosome in males to mediate hypertranscription (Gorman *et al.* 1995). A dosage compensation defect does not appear to be the direct cause of hybrid lethality because X chromosome transcripts are not preferentially affected in lethal hybrids (Wei *et al.* 2014). However, several observations suggest that dosage compensation genes may interact with hybrid lethality. Components of the DCC fail to localize to the X chromosome and H4K16Ac is not enriched on the X chromosome in hybrid males compared with pure species males (Pal Bhadra *et al.* 2006), although a later study did detect Msl-2 protein on the X (Thomae *et al.* 2013). Additionally, mutations in *D. melanogaster* DCC genes, including *msl-3*, mildly enhance hybrid male viability when partially rescued by *Lhr* alleles (Barbash 2010a).

### siRNA was ineffective in silencing in *D. simulans msl-3*

We first tested *D. melanogaster msl-3* as a candidate responsible for rescue by crossing *msl-3^1^* females to *D. mauritiana*. No live or pharate hybrid males were observed (270 and 84 *msl-3^1^*/+ hybrid females recovered in crosses with *D. mauritiana* iso 105 and W139, respectively). We next attempted to test *sim-msl-3* by knocking down its expression in hybrids using RNAi. The experimental challenge was to design an RNAi construct that would target the *D. simulans* ortholog but not the *D. melanogaster* ortholog, because removing both copies of *msl-3* would result in male lethality. The shmiR method seemed ideal because it can achieve potent knockdown by expressing a single 21-bp siRNA from a modified miRNA-based vector (Haley *et al.* 2008). We used the Valium 20 transformation vector, which expresses under the control of upstream activation sequence (UAS) sequences when crossed to a strain expressing the Gal4 activator protein (Ni *et al.* 2011). Our strategy was to transform these RNAi constructs into the *attP2* site on *D. melanogaster* chromosome 3 and then cross transformed stocks to a strain containing *actin-Gal4* on chromosome 2. These flies will express ubiquitously the shmiR, and when females are crossed to *D. simulans* males, one-quarter of the F1 hybrid progeny will inherit both the *Gal4* driver and the *UAS*-driven shmiR.

**Figure 4:**
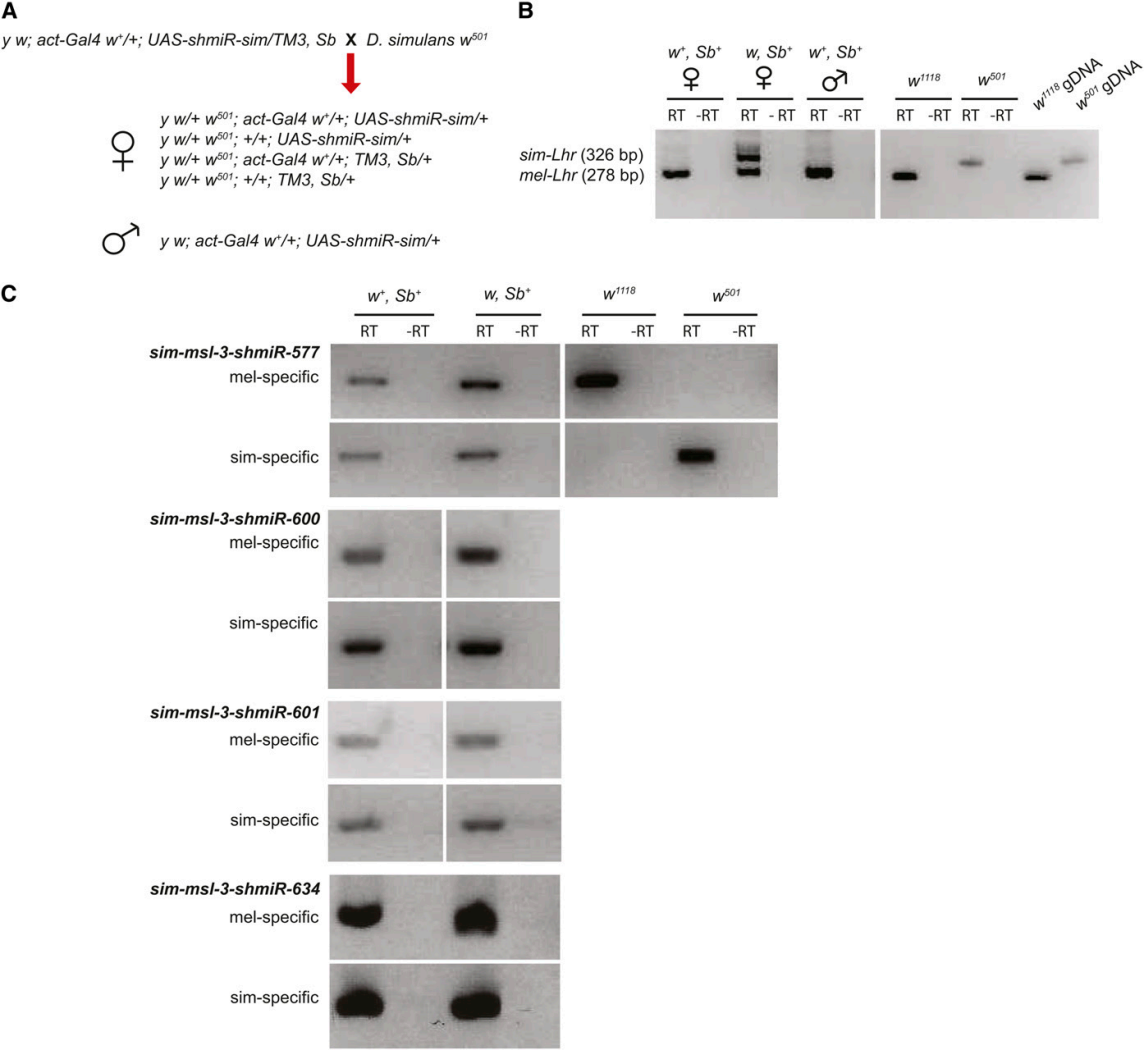
RT-PCR tests of shmiR knock-down of *D. simulans Lhr* and *msl-3*. (A) *D. melanogaster* females expressing either *Lhr* or *msl-3* siRNAs targeting the *D. simulans* orthologs (abbreviated as *UAS-shmiR-sim*) were crossed to *D. simulans* males. *w^+^*, *Sb^+^* hybrid progeny (1/4 of the total) inherit both the *Gal4* driver and *UAS-shmiR*. The males will survive if the *UAS-shmiR* construct knocks down expression of a hybrid lethality gene. (B and C) RT-PCR tests of knockdown. (B) Hybrid male and female progeny carrying both *act-Gal4* and *UAS-shmiR-sim-Lhr* (*w^+^*, *Sb^+^*) express *D. melanogaster mel-Lhr* (278 bp) but not *sim-Lhr* (326 bp), assayed using a single primer pair that detects an insertion in *sim-Lhr*. Hybrid females carrying only *UAS-shmiR-sim-Lhr* (*w*, *Sb^+^*) were used as a control and express both orthologs. Right panel is RT-PCR and genomic DNA (gDNA) controls from *D. melanogaster w^1118^* and *D. simulans w^501^*. (C) None of four tested *sim-msl3-shmiR* constructs silence *sim-msl-3* expression in hybrid female progeny. Separate PCRs were performed using primer pairs specific to either *mel-msl-3* or *sim-msl-3*, as confirmed using controls as described previously. Hybrid females carrying both *act-Gal4* and *UAS-shmiR-sim-msl-3* (*w^+^*, *Sb^+^*) expressed both *msl-3* orthologs. As a control, progeny only inheriting *UAS-shmiR-sim-msl-3* (*w*, *Sb^+^*) were also assayed (except for *sim-msl-3-shmiR-577* where both *w*, *Sb^+^* and *w*, *Sb* animals were pooled), and expressed both orthologs as expected. RT-PCR, reverse-transcription polymerase chain reaction.

As a positive control, we first designed a vector that targets an insertion that is specific to *D. simulans Lhr (sim-Lhr)* (Table 1 and Table 5). Expression of this construct in *D. melanogaster* produced no phenotype. All four expected genotypes of F1 hybrid females were recovered. RT-PCR analysis showed that only *mel-Lhr* is expressed in *act-Gal4/+*; *UAS-shmIR-sim-Lhr/+* whereas both orthologs are expressed in control hybrid females (*+/+*; *UAS-shmIR-sim-Lhr/+*), demonstrating that the construct specifically knocks down *sim-Lhr* expression (Figure 4B). Interestingly, at 25° females expressing the shmiR against *sim-Lhr* had the greatest viability, which is consistent with *sim-Lhr* having a dominant effect on hybrid female viability at greater temperatures (Barbash *et al.* 2000). As predicted, knockdown of *sim-Lhr* rescued hybrid males, and RT-PCR analysis again demonstrated specific knockdown of the *sim-Lhr* otholog (Figure 4).

**Table 5:** Suppression of hybrid lethality by *UAS-shmIR-sim-Lhr*

Temperature	Sex of Progeny	*GAL4/+*; *UAS/+* (w^+^ Sb^+^)	*GAL4/+*; *+/+* (w^+^ Sb)	*+/+*; *UAS/+* (w Sb^+^)	*+/+*; *+/+* (w Sb)
25°	Female	217	32	165	47
	Male	144	0	0	0
18°	Female	62	79	58	57
	Male	58	0	1	0

*y w; P{w[+mC]=Act5C-GAL4}25FO1 /+; φ{UAS-shmIR-sim-Lhr}attP2, v^+^ y^+^/TM3,Sb D. melanogaster* females were crossed to *w^501^ D. simulans* males. The transgenes are abbreviated as “*GAL4*” and “*UAS*” in the table headings. Number of progeny of the indicated genotype (phenotype) are listed.

We designed seven different shmiRs against *sim-msl-3* that each had mismatches to *mel-msl-3* (Table 1). Some were designed with modifications from published schemes (see the section *Materials and Methods*). Three of the constructs were lethal or semilethal to both sexes when expressed in *D. melanogaster* (Table 1), which must be due to off-target effects because *msl-3* is only required in males. A fourth construct (*msl-3-shRNA-600*) was lethal only to *D. melanogaster* males. Because *msl-3* is expressed in females, we could assay the effect of this shmiR, and found by RT-PCR that it does not reduce *msl-3* expression (Figure 4C). Therefore, this construct likely has a post-transcriptional effect on *mel-msl-3*.

The remaining three constructs were viable within *D. melanogaster* and thus could be crossed to *D. simulans* as we did for the *Lhr* control. None produced any hybrid males, but RT-PCR demonstrated that none silenced *sim-msl-3* expression. We were thus unable to test whether *sim-msl-3* affects hybrid male viability.

### *mel-Hmr* does not cause lethality to *X_sim_* hybrid males

Our screen was limited to the autosomes, but we wished to test whether *D. melanogaster (mel-Hmr)* can account entirely for the lethal effect of *X_mel_* in hybrid males. *X_sim_* (and *X_mau_*) hybrid males are viable (Sturtevant 1920; Hutter *et al.* 1990), and a simple prediction is that the presence of *mel-Hmr* will kill *X_sim_* hybrid males if *mel-Hmr* is the sole X-linked difference between these species involved in hybrid lethality. Hybrid sons carrying the paternal X chromosome can be generated by crossing *D. simulans* males to compound-X *D. melanogaster* females. We tested the role of *mel-Hmr* by using a *mel-Hmr-HA* transgene (Satyaki *et al.* 2014) and found that it had no effect on *X_sim_* hybrid male viability (Table 6). We conclude that additional genes and/or sequences on *X_mel_* are required for the fully penetrant lethality of *X_mel_* hybrid males.

**Table 6:** A *mel-Hmr* transgene does not reduce *X_sim_* hybrid male viability

Hybrid Females		Hybrid Males		
*C(1)DX*; *+/+*	*C(1)DX*; *ø{mel-Hmr-HA}/+*	*w/Y*; *+/+*	*w/Y*; *ø{mel-Hmr-HA}/+*	Relative Viability
0	0	132	108	81.8%*^a^*

*C(1)DX, y w f/Y; ø{mel-Hmr-HA}/+ D. melanogaster* females were crossed to *w^501^/Y D. simulans* males at 22-23°.

a Not significant by χ^2^ test (*P* > 0.05).

## Discussion

### Major-effect *vs.* minor-effect HI genes

We used the Bloomington Deficiency Kit to screen for dominant suppressors of lethality in interspecific hybrids between *D. melanogaster* and *D. mauritiana*. Our screen is different from previous screens (Coyne *et al.* 1998; Presgraves 2003; Matute *et al.* 2010) in that it is designed to identify putative HI loci that cause dominant lethality, like *Hmr* and *Lhr*. The removal of either *Hmr* or *Lhr* suppresses hybrid male lethality, which classifies them as major-effect HI genes. Because we only screened for hybrid rescue with *D. mauritiana*, our screen also is predicated on the assumption that additional HI loci will be similar to *Hmr* and cause lethality with all three sibling species of *D. melanogaster*.

Our failure to observe any adult males suggests that the *D. melanogaster* genome does not harbor additional major-effect HI loci within the regions screened, but this does not exclude the possibility that there are additional factors of minor effect that contribute to hybrid lethality. We identified four regions, in addition to the region on 2R containing *Lhr*, which when deleted rescue hybrid male lethality to the pharate stage, suggesting the presence of minor-effect HI loci. However, the weak nature of these rescuing effects makes them susceptible to suppression by background effects. Without the ability to confirm them with multiple overlapping deficiencies, we conclude that the rescuing deficiencies identify regions that can be tentatively considered to contain minor-effect hybrid lethality loci.

The regions deleted by *Df(3L)BSC27* and *Df(3L)BSC33* gave the strongest level of rescue. Surprisingly, these two deletions are distinct and do not overlap, as predicted by estimated cytological breakpoints and confirmed by complementation crosses and sequencing. It is possible, however, that both deletions affect a single gene. There are ~93 kb between *Df(3L)BSC27* and *Df(3L)BSC33*. Most enhancers in *Drosophila* are within 10 kb of their target sequence, but longer-range interactions are known (Berman *et al.* 2004). For example, the *cut* gene is regulated by an enhancer 80 kb upstream of its promoter (Jack and Delotto 1995). A recent genome-wide study estimated that ~28% of enhancers are >20 kb from their targets and can be more than 100 kb away (Kvon *et al.* 2014).

Pontecorvo’s experiments (1943) suggest that gene(s) on the *D. simulans* 3rd chromosome contribute to hybrid lethality. Based on previous findings that *D. melanogaster Lhr* has a weak effect on hybrid lethality, we reasoned that our screen has the ability to identify major-effect genes in *D. simulans* by detecting weak effects of deleting the *D. melanogaster* ortholog. We attempted to test *msl-3* as one such candidate by knocking down expression of *sim-msl-3* using shmiRs but unfortunately failed to do so.

### Challenges with using shmiRs to knockdown gene expression

The shmiR system is attractive because the expression of a single siRNA allows the design of siRNAs that target only one of the two orthologs in hybrids. We were successful in specifically targeting *sim-Lhr* by designing a shmiR targeting a species-specific insertion in the *sim-Lhr* coding sequence. However, we were unable to achieve specific knockdown of *sim-msl-3*. Three constructs caused lethality to both sexes within *D. melanogaster* (Table 1), which must be off-target effects as *msl-3* is only required in males. One shmiR (*msl-3-sim-shmiR-600*) was lethal in *D. melanogaster* males, even though it has a mismatch to the *D. melanogaster msl-3* ortholog near its center. We found that *msl-3* mRNA expression is not reduced in the lethal males, suggesting that *msl-3* translation is likely being blocked via a microRNA-like effect of the seed sequence. Because as little as 6 bp of sequence homology near the 5′ end of a microRNA can be sufficient for its activity (Lewis *et al.* 2005), this hypothesis would explain why the shmiR designed to target *sim-msl-3* would be able to target the *D. melanogaster* ortholog. This type of off-target effect will be challenging to predict because it requires limited homology and suggests the need for caution when expressing shmiRs at a high level. The clustered regularly interspaced short palindromic repeats (CRISPR/Cas9) system may prove an effective alternative in the future for targeting mutations to *D. simulans* candidate HI genes.

### The potential role of X-chromosome genes

The X chromosome contains 15% of *D. melanogaster* genes (Adams 2000), but we could not screen it because deletions of the X are lethal in males. We showed here that *mel-Hmr* is insufficient to explain the X-linked portion of hybrid male lethality, because a *mel-Hmr-HA* transgene does not induce lethality of *X_sim_* hybrid males (Table 6). This result is consistent with previous findings that a similar transgene significantly reduces viability of *X_mel_*/*X_sim_* hybrid females but does not induce full lethality (Barbash *et al.* 2003), in contrast to the invariant lethality of *X_mel_*/*X_mel_* hybrid females (Hutter *et al.* 1990).

*Df(1)307-1-2* identifies a candidate region in 9D that is adjacent to but nonoverlapping with *Hmr* (Barbash *et al.* 2003), but the causal gene remains unidentified. A recent screen examined *Y*-linked duplications of *X_mel_* regions for lethality in *X_sim_* hybrid males and identified 2 candidate regions (Matute and Gavin-Smyth 2014). One of these regions (9C-10B) is not a new discovery, as *Dp(1;2)v^+75d^* covering 9A2-10C2 was previously shown to reduce *X_mau_*, *X_sec_*, and *X_sim_* hybrid male viability (Barbash *et al.* 2000; Orr and Irving 2000). The duplication also significantly reduces *X_mel_*/*X_mau_* and *X_mel_*/*X_sim_* hybrid female viability (Barbash *et al.* 2000), presumably due at least in part to the aforementioned effect of *Hmr*. This effect in females demonstrates that the 9C-10B duplication causes dominant lethality by interacting with *D. simulans* and *D. mauritiana* genes that are also dominantly acting and could be autosomal or X-linked, in contrast to the conclusion that it represents a dominant-recessive interaction between the X chromosomes (Matute and Gavin-Smyth 2014).

We have shown that *Hmr* cannot be the sole cause of the duplication-induced hybrid male lethality (Table 6), although it may be contributing. We suggest that lethality may result from the cumulative dosage increase of multiple duplicated genes, because well-characterized HI genes act as gain-of-function alleles in a hybrid background (Maheshwari and Barbash 2011). These putative dosage effects can only be tested for in a hybrid background. We reiterate here that testing fitness effects of chromosome aberrations within *D. melanogaster* is a useful general control but has no bearing on whether or not duplications or deficiencies are responsible for dosage effects in hybrids, because HI genes by definition have distinct (and often opposite) properties in hybrid *vs.* pure species backgrounds (Maheshwari and Barbash 2011).

## Supplementary Material

Supporting Information
