# Supplementary material for: A Screen for F1 Hybrid Male Rescue Reveals No Major-Effect Hybrid Lethality Loci in the Drosophila melanogaster Autosomal Genome
Source: G3 (Bethesda). 2014 Oct 27;4(12):2451–60. doi: 10.1534/g3.114.014076 (PMC4267940; doi:10.1534/g3.114.014076)
Supplement: Supporting Information [file supp_4_12_2451__index.html]

A Screen for F1 Hybrid Male Rescue Reveals No Major-Effect Hybrid Lethality Loci in the Drosophila melanogaster Autosomal Genome — Supporting Information 

# A Screen for F1 Hybrid Male Rescue Reveals No Major-Effect Hybrid Lethality Loci in the *Drosophila melanogaster* Autosomal Genome

## Supporting Information for Cuykendall *et al.*, 2014

**Files in this Data Supplement:**

- Supporting Information - Tables S1-S2 (PDF, 145 KB
- Table S2 - 3L complementation crosses. (PDF, 132 KB)
- Table S1 - Hybrid viability data from all interspecific crosses. (.xlsx, 119 KB)
