# Supplementary material for: A Screen for F1 Hybrid Male Rescue Reveals No Major-Effect Hybrid Lethality Loci in the Drosophila melanogaster Autosomal Genome
Source: G3 (Bethesda). 2014 Oct 27;4(12):2451–60. doi: 10.1534/g3.114.014076 (PMC4267940; doi:10.1534/g3.114.014076)
Supplement: Supporting Information [file supp_g3.114.014076_TableS2.pdf]

Table S2 3L complementation crosses. Complementation was observed between deficiencies if progeny were viable. Complementation with *msl-3* was indicated by the rescue of male lethality. Both *Df(3L)BSC27* and *Df(3L)Exel6110* do not complement *msl-3*.

Deficiency 1			Deficiency 2			Complements?
Name	Estimated breakpoints		Name	Estimated breakpoints		
msl-3	7116313	7118633	*Df(3L)Exel6110*	7087906	7149284	**No**
msl-3	7116313	7118633	*Df(3L)RM5-1*	6999777	7287396	Yes
Df(3L)BSC27	6963120	7132538	*Df(3L)Exel6110*	7087906	7149284	**No** [a]
Df(3L)BSC27	6963120	7132538	*Df(3L)BSC33*	7271620	7319021	Yes
Df(3L)BSC27	6963120	7132538	*Df(3L)W5.4*	5919622	7029849	Yes
Df(3L)BSC27	6963120	7132538	*Df(3L)XDI98*	5967841	7029849	Yes
Df(3L)BSC27	6963120	7132538	*msl3*	7116313	7118633	**No**
Df(3L)RM5-1	6999777	7287396	*Df(3L)BSC27*	6963120	7132538	Yes
Df(3L)RM5-1	6999777	7287396	*Df(3L)BSC33*	7271620	7319021	Yes
Df(3L)Exel6110	7087906	7149284	*Df(3L)RM5-1*	6999777	7287396	Yes
Df(3L)BSC33	7271620	7319021	*Df(3L)BSC27*	6963120	7132538	Yes
Df(3L)BSC33	7271620	7319021	*Df(3L)RM5-1*	6999777	7287396	Yes
Df(3L)BSC33	7271620	7319021	*Df(3L)Exel6110*	7087906	7149284	Yes
Df(3L)W5.4	5919622	7029849	*Df(3L)Exel6110*	7087906	7149284	Yes
Df(3L)W5.4	5919622	7029849	*Df(3L)RM5-1*	6999777	7287396	Yes
Df(3L)XDI98	5967841	7029849	*Df(3L)Exel6110*	7087906	7149284	Yes
Df(3L)XDI98	5967841	7029849	*Df(3L)RM5-1*	6999777	7287396	Yes
Df(3L)BSC374	6957558	7032145	*Df(3L)W5.4*	5919622	7029849	Yes
Df(3L)BSC374	6957558	7032145	*Df(3L)BSC27*	6963120	7132538	**No**
Df(3L)BSC374	6957558	7032145	*Df(3L)Exel6110*	7087906	7149284	Yes
Df(3L)BSC224	6957557	7150109	*Df(3L)W5.4*	5919622	7029849	Yes

[a] Stocks essentially did not complement. Only 4 homozgous deficiency-carrying progeny compared to 100 heterozygous progeny.
